# Uncompleted emergency department care and discharge against medical advice in patients with neurological complaints: a chart review

**DOI:** 10.1186/s12873-019-0273-y

**Published:** 2019-10-11

**Authors:** Carolin Hoyer, Patrick Stein, Angelika Alonso, Michael Platten, Kristina Szabo

**Affiliations:** Department of Neurology, Medical Faculty Mannheim, Universitätsmedizin Mannheim, Heidelberg University, Theodor-Kutzer-Ufer 1-3, 68135 Mannheim, Germany

**Keywords:** Neurology, Emergency department, Patient discharge

## Abstract

**Background:**

Uncompleted emergency department care and against-medical-advice discharge represent relevant medical problems with impact on patient safety and potential medicolegal and socioeconomic consequences. They may also indicate structural or procedural problems in the emergency department (ED) relating to patient management and flow. While patients with neurological complaints frequently leave the ED against medical advice or without being seen, no dedicated analysis of this group of patients aiming at the identification of characteristics associated with irregular ED discharge has been performed so far.

**Methods:**

A chart review was performed of all patients with neurological complaints presenting to a German interdisciplinary emergency department between January and December 2017 for neurological evaluation. Demographics, mode of presentation, process times, presenting symptoms and diagnosis were recorded. Patients leaving against medical advice after an informed consent discussion and signing of documentation (DAMA) or leaving prematurely without notifying ED staff (PL) were compared to the total of patients who were admitted or discharged (non-DAMA/PL).

**Results:**

Of all patients presenting with neurological symptoms or complaints, 3% left against medical advice and 2.2% left prematurely. DAMA/PL patients were younger (*p* < .001), and they were more frequently self-presenting (*p* < 0.001). Headaches, seizures and sensory deficits were the most frequent presenting symptoms in DAMA/PL patients, and 56.1% of those presenting with a seizure had a history of epilepsy. The most common documented reason for leaving was the duration of door-to-doctor time.

**Conclusions:**

Younger age, self-presenting mode of presentation and presentation with headache, seizures or sensory deficits are associated with premature leave or against-medical-advice discharge of patients with neurological complaints from the ED, and long waiting times were given as the major reason for leaving the ED. Increasing ED staff’s awareness of these factors and the optimization of pre-hospital assessment and demand management, thereby positively impacting on patient flow and ED process times, may help to prevent irregular discharges from the ED.

## Background

- Discharge against medical advice (DAMA), uncompleted emergency care and emergency department (ED) walk-outs, often without being seen by a medical professional (LWBS), occur in 1–3% of ED visits [1]. They present relevant and multi-faceted problems: With an increased risk for adverse health events, ED readmission and subsequent hospitalization [2–4], DAMA and LWBS negatively impact patient safety, and also carry medicolegal and socioeconomic implications [5–7]. Previous studies have identified a number of predictors for DAMA or LWBS. Besides male sex, younger age, low socioeconomic status, and substance abuse [8], other relevant factors include triage category, mode and time of arrival. Presentation during periods of high patient traffic has been found to increase the odds of patients leaving the ED prematurely, presumably due to longer waiting times [9–11]. Door-to-doctor and other process times critically influence patient throughput, which is a major quality indicator for ED patient management [12]. Hence, a high prevalence may indicate that an institution’s ED patient flow and management requires review and improvement.
- Patients presenting with neurological complaints in the ED are often particularly challenging because signs or symptoms may be non-specific and the ensuing diagnostic and therapeutic implications may not be evident from the outset [13, 14]. For example, headache, the most common chief complaint among ED patients with neurological complaints [15], is frequently found among patients leaving against medical advice [1, 16]. While most headaches are of benign etiology, a small percentage of patients have a serious underlying cause warranting immediate medical attention [17]. In addition, neurological symptoms may manifest episodically in non-benign conditions, such as the waxing and waning course observable in patients with basilar artery thrombosis. This may theoretically lead patients to prematurely leave the ED due to intermittent symptom improvement and subsequent clinical deterioration may be brought to medical attention with relevant delay and potentially devastating consequences. To date, studies about DAMA and PL from the ED have not specifically focused on patients with neurological complaints, who have been identified to be at a high risk of leaving the ED prior to completion of care [1, 18]. In a retrospective analysis, we sought to investigate the characteristics of patients admitted to our interdisciplinary ED for neurological evaluation in order to identify factors associated with DAMA or uncompleted ED care in this group of patients compared to patients who were admitted or discharged.

## Methods

### Study design

We retrospectively analyzed patient records of patients who consecutively presented or were referred to the interdisciplinary ED of the University Medical Centre, Mannheim, Germany, between January 1st 2017 and December 31st 2017 for neurological consultation. The University Medical Centre is a full-service healthcare institution providing comprehensive care for inpatients and outpatients from the city of Mannheim (current population: approximately 310.000) and surroundings. In the interdisciplinary ED, physicians from different specialties are present, and at least one neurology resident is available 24/7 either for first-line assessment if prehospital evaluation suggests a neurological condition, or second-line as per judgement of a non-neurologist ED physician upon patient arrival. Cases were identified by filtering the ED database for patients coded to present with a chief complaint that was evaluated by emergency medical service or ED staff upon arrival to be a neurological symptom or complaint. ED documentation of all of these patients was checked for details regarding the presenting symptom and diagnosis at discharge, if given, as well as for discharge modality. Patients were categorized as discharged against medical advice (DAMA) when they received an explanation about risks and consequences by an ED physician at any time during their stay and signed a standardized DAMA form. We defined premature leave (PL) patients as those who left without notifying ED staff of their intention to leave and accordingly without an informed consent discussion and the signing of respective documentation. This also includes LWBS patients. All data that were analyzed in this study were extracted from patient records relating to their stay in the ED. This also included the evaluation as to whether ED work-up was complete, which was the case when all examinations deemed necessary by the ED neurologist for making a diagnosis were completed. The study was approved by the local ethics committee.

### Statistical analysis of neurological referrals to the interdisciplinary ED

Data analyzed included basic demographic information, whether the patient lived in the local area (living within a perimeter of 25 km from the hospital) or outside of the local area, or who was without a place of residence or not living in Germany (subsumed as “other”), chief complaint/presenting symptom according to Royl et al. [15], ED discharge diagnosis if given, time and mode of presentation to the ED, door-to-doctor time and length of ED stay. In DAMA/PL patients, the reason for DAMA/PL if documented, was obtained from medical records.

Since DAMA/PL patients left at different stages of diagnosis or treatment, it was often unclear whether a complete evaluation would have resulted in recommendation for admission or discharge. Consequently, we compared characteristics of DAMA/PL patients and all patients who were either admitted or discharged (=non-DAMA/PL).

Statistical analysis was performed with STATA for Mac, StataCorp. 2017. Stata Statistical Software: Release 15. College Station, TX: StataCorp LLC. The Kolmogorov-Smirnov goodness-of-fit test was used to test whether the variables such as basic demographic information, door-to-doctor time and length of ED stay were normally distributed. As the data were not normally distributed, non-parametric tests were used. The distribution of categorial variables between groups was compared by chi2 tests or Fisher’s exact test, depending on group sizes. Group comparisons were assessed using Wilcoxon-Mann-Whitney test. Bonferroni correction for multiple testing was applied where suitable. A *p*-value of <.05 was considered significant.

## Results

### Disposition and demographics

Within the 12-month period under consideration, a total of 45,445 patients were seen in the ED. In 5340 (12.0%) of these, a neurological evaluation was requested. Of all patients with neurological complaints, 2530 (47.4%) were admitted, 2529 (47.4%) were discharged. One-hundred and sixty-one patients (3.0%) were discharged against medical advice (=DAMA), and 120 patients (2.2%) left without informing ED staff (=PL).

Mean age of patients with neurological complaints was 56.2 years (SD ± 21.27), 2583 (48.4%) were male. While there were no differences in gender distribution, mean age and age spectra differed significantly between DAMA/PL and non-DAMA/PL patients with DAMA/PL patients being significantly younger (*p* < 0.001; Table 1). Moreover, patients without a place of residence or living in a foreign country were more likely to leave DAMA/PL (*p* < 0.001).

**Table 1:** Characteristics of patients leaving prematurely or against medical advice and patients regularly discharged or admitted

	Patients who signed to leave against medical advice (*N* = 161) or left before complete ED care without signing (*N* = 120); total *N* = 281	Patients who were discharged or admitted (*N* = 5059)	*p* value*
*Demographics*			
age, mean (SD; IQR)	44.1 (19.2; 28–57)	56.9 (21.2; 39–76)	**< 0.001**
< 30, N (%)	84 (29.9%)	825 16.3%)	**< 0.001**
30–50, N (%)	94 (33.5%)	1104 (21.8%)	**< 0.001**
50–70 N (%)	71 (25.3%)	1456 (28.8%)	0.205
> 70, N (%)	32 (11.4%)	1674 (33.1%)	**< 0.001**
sex, M, N (%)	141 (50.2%)	2442 (48.3%)	0.535
living in local area, N (%)	174 (61.9%)	2986 (59.0%)	0.336
living outside local area, N (%)	93 (33.1%)	1985 (39.2%)	0.040
other, N (%)	14 (5.0%)	88 (1.7%)	**< 0.001**
*Time of ED presentation, N (%)*			
weekend	73 (26.0%)	1220 (24.1%)	0.478
0 h–6 h	22 (7.8%)	475 (9.4%)	0.381
6 h–12 h	63 (22.4%)	1425 (28.2%))	0.036
12 h–18 h	129 (45.9%)	1981 (38.9%)	0.024
18 h–24 h	67 (23.8%)	1178 (23.3%)	0.829
*ED times in min, mean (SD; IQR)*			
door-to-doctor time	43.0 (78.3; 8–36)	38.7 (67.0; 6–40)	0.32
ED length of stay	264.6 (311.0; 140–320)	260.4 (173.6; 132–347)	0.69
*Mode of presentation, N (%)*			
Self-presenting	140/236 (59.3%)	2055/5055 (40.7%)	**< 0.001**
Emergency medical service (EMS)	75/236 (31.8%)	2481/5055 (49.1%)	**< 0.001**
EMS with emergency physician	21/236 (8.9%)	518/5055 (10.2%)	0.503
*Presenting symptom according to Royl, 2010, N (%)*			
Ataxia/movement disorder	2/221 (0.9%)	60 (1.2%)	1.000
Impaired consciousness	7/221 (3.2%)	238 (4.7%)	0.288
Seizure	41/221 (18.6%)	560 (11.1%)	**0.001**
Headache	36/221 (16.3%)	652 (12.9%)	0.141
Other pain	8/221 (3.6%)	139 (2.7%)	0.440
Motor deficit	20/221 (9.0%)	652 (12.9%)	0.094
Confusion/amnesia	7/221 (3.2%)	279 (5.5%)	0.131
Disturbed vision	17/221 (7.7%)	254 (5.0%)	0.078
Sensory deficit	34/221 (15.4%)	426 (8.4%)	**< 0.001**
Impaired language/speech/ swallowing	12/221 (5.4%)	491 (9.7%)	0.034
Vertigo	31/221 (14.0%)	776 (15.3%)	0.596
Other neurological complaint	1/221 (0.5%)	282 (5.5%)	**< 0.001**
Non-neurological complaint	5/221 (2.3%)	250 (4.9%)	0.076

*With Bonferroni correction (for 10 tests): statistical significance with *p* < 0.005

### Time and mode of presentation

Patients presenting during daytime were more likely to leave DAMA/PL (Table 1). Door-to-doctor times did not differ between the groups, but duration of stay in the ED was significantly shorter in DAMA/PL patients (*p* < 0.001; Table 1). DAMA/PL patient were significantly more frequently self-presenting (*p* < 0.001; Table 1).

### Presenting symptoms

Percentages of frequencies of presenting symptoms categorized by DAMA/PL vs. non-DAMA/PL are presented in Table 1. Most frequent presenting symptoms in DAMA/PL patients were headaches, seizures and sensory deficits. Seizures (*p* = 0.001) and sensory deficits (*p* < 0.001) occurred significantly more frequently in DAMA/PL patients, while impairments of language, speech or swallowing were found more frequently in non-DAMA/PL patients (*p* < 0.034).

### Detailed description of DAMA/PL patients

**Fig. 1:**
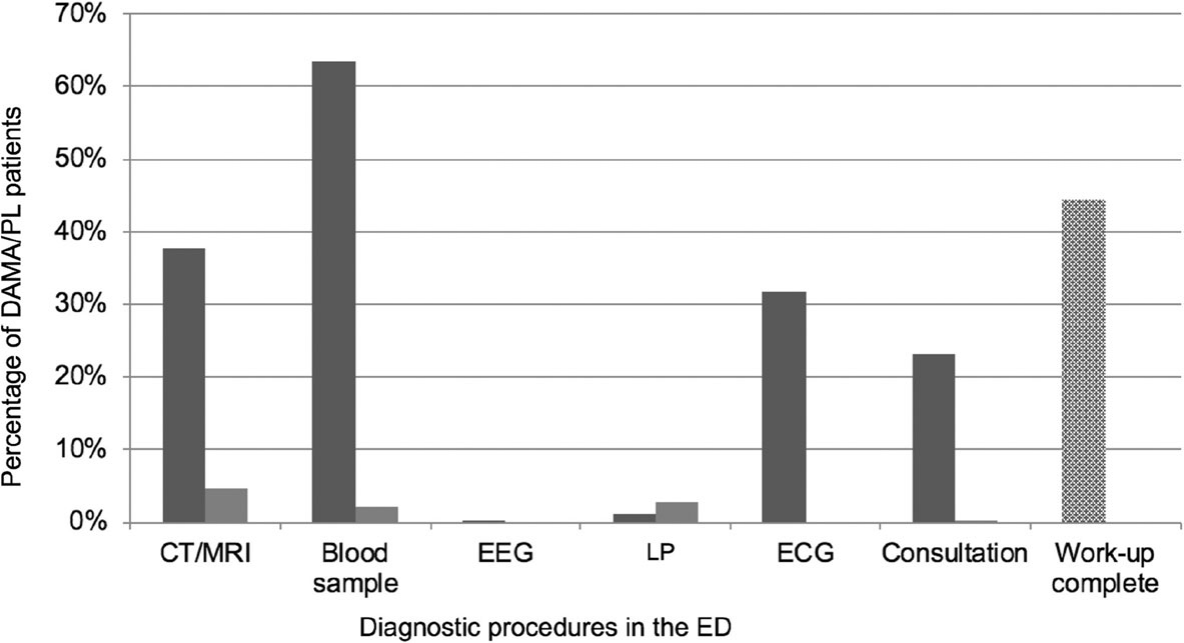
Diagnostic procedures in DAMA/PL patients. Dark grey: performed procedures, %. Light grey: declined procedures, %. Dotted: Proportion of DAMA/PL patients with complete diagnostic work-up, %

Of all 281 DAMA/PL patients, 156 (55.5%) left prior to physical exam or diagnostic measures deemed necessary for further evaluation. One-hundred and twenty-five patients (44.5%) left after complete ED diagnostic work-up yielding an indication for admission, one of these patients left after complete work-up but did not inform ED staff of their intention to leave. Figure 1 depicts the proportions of diagnostic procedures in DAMA/PL patients that were performed or recommended but declined by the patient as well as the proportion of patients in whom a complete work-up was performed. In 51 cases, the reason for DAMA/PL was documented: 36 patients stated the need to wait for the physician to begin the examination as reason to leave, mean door-to-doctor times in these patients was 97.7 (±106.2, IQR: 24–148) mins, which was indeed significantly longer than all other patients (58.1 ± 85.7; IQR: 11–63; *p* = 0.006). Other reasons given were: symptom improvement either spontaneously (two patients) or because of analgesia administered in the ED (one patient), need to care for family members (six patients), preferred treatment in a different hospital closer to home (four patients), need to go to work and other urgent appointment (one patient each). A comparison of patients leaving the ED prior to and after complete ED work-up is given in Table 2.

**Table 2:** Comparison of demographics, mode of presentation and presenting symptom between patients who left prior to and after completion of ED diagnostic work-up

	Patients who left the ED before the ED diagnostic work-up was complete (*N* = 156)	Patients who left the ED after complete ED diagnostic work-up (*N* = 125)	*p* value*
*Demographics*			
age, mean (SD; IQR)	40.6 (17.1; 27–53)	47.5 (20.2; 30–61)	0.005
sex, M, N (%)	72 (46%)	70 (56%)	0.12
*ED times in mins, mean (SD; IQR)*			
door-to-doctor time	81.5 (96.0; 15–122)	41.8 (74.4; 8–40)	**< 0.001**
ED length of stay	116.8 (159.5; 0–199)	275.2 (351.9; 147–321)	**< 0.001**
*Mode of presentation, N (%)*			
Self-presenting	88/125 (73%)	61 (49%)	0.09
Emergency medical service (EMS)	24/125 (20%)	51 (41%)	**< 0.001**
EMS with emergency physician	8/125 (7%)	13 (10%)	0.18
*Presenting symptom according to Royl, 2010, N (%)*			
Ataxia/movement disorder	2/112 (1.8%)	1 (0.8%)	1.00
Impaired consciousness	3/112 (2.7%)	5 (4.0%)	0.47
Seizure	21/112 (18.8%)	23 (18.4%)	0.32
Headache	32/112 (28.6%)	10 (8.0%)	0.004
Other pain	6/112 (5.4%)	2 (1.6%)	0.31
Motor deficit	4/112 (3.6%)	17 (13.6%)	**0.001**
Confusion/amnesia	3/112 (2.7%)	5 (4.0%)	0.47
Disturbed vision	8/112 (7.1%)	10 (8.0%)	0.46
Sensory deficit	11/112 (9.8%)	23 (18.4%)	0.005
Impaired language/speech/swallowing	–	12 (9.6%)	**< 0.001**
Vertigo	20/112 (17.9%)	13 (10.4%)	0.58
Other neurological complaint	–	1 (0.8%)	0.45
Non-neurological complaint	2/112 (1.8%)	3 (2.4%)	0.66

*With Bonferroni correction (for 21 tests): statistical significance with *p* < 0.002

Discharge diagnoses of DAMA/PL patients are listed in Table 3. While in 61 cases (21.7%), no discharge diagnosis could be arrived at, most frequent diagnoses in DAMA/PL patients were seizures and headaches. In 23 of 41 (56.1%) DAMA/PL cases with seizure as presenting symptom, epilepsy was known and previously diagnosed.

**Table 3:** Discharge diagnoses of DAMA/PL patients in alphabetical order. Insufficient information prevented making a diagnosis in 61 cases

Discharge diagnosis of DAMA/PL patients (in alphabetical order)	Frequency, *N* (%)
CNS demyelinating autoimmune disease	6 (2.14)
CNS tumour	1 (0.36)
Intracranial hemorrhage	2 (0.71)
Manifest ischemic stroke	11 (3.91)
Migraine and other headache	40 (14.23)
Movement disorder	6 (2.14)
Non-neurological disorder	35 (12.46)
Other neurological disorder	28 (9.96)
Peripheral nerve palsy	6 (2.14)
Seizure	44 (15.66)
Transient ischemic attack	17 (6.05)
Vertigo	24 (8.54)

## Discussion

- We retrospectively studied records of patients discharged against medical advice or premature leave initially presenting with neurological complaints to an interdisciplinary ED, aiming to identify factors associated with irregular discharge in this group of patients. This issue has hitherto not been investigated in detail. We found a DAMA rate of 3% and a PL rate of 2%, which in addition to the observed predominance of younger age in the DAMA/PL subgroup is comparable to studies of unselected ED patient populations [1, 18, 19].
- Neurological conditions have been observed to carry a high risk of DAMA [18]. Headaches, seizures and sensory deficits were the most frequent presenting symptoms in DAMA/PL patients in our investigation, and the latter two were significantly more prevalent in DAMA/PL compared to non-DAMA/PL patients. In our study population, more than half of DAMA/PL patients presenting with a seizure had a previously known epilepsy. Patients with known epilepsy and – in retrospect – typical seizures make up a considerable portion of ED admissions due to seizures [20]. Limited access to relevant information on scene regarding whether a seizure was typical or whether it may have been secondary to some condition requiring immediate medical attention as well as the lack of formal non-conveyance criteria often impact the decision of emergency medical service (EMS) staff to err on the side of safety and transport a patient to hospital [21, 22]. As a consequence, incongruencies regarding the perceived necessity for ED presentation between patients and EMS or ED personnel may be one factor contributing to DAMA/PL in this subgroup of patients. Such differences in perception and evaluation may also underlie the higher proportion of patients with sensory deficits leaving DAMA/PL. While they may indicate a serious underlying pathology, it can be hypothesized that sensory deficits are less functionally impeding or less noticeable to other people than neurological deficits such as dysarthria, motor deficits or gait ataxia. Accordingly, patients with sensory deficits may wish to leave the ED despite the need for further in-hospital work-up or monitoring. The large proportion of DAMA/PL patients presenting with headache, which also rated among the top ten DAMA diagnoses in a general population [1], presumably reflects – at least in part – the fact that headache, in most cases in the context of a primary headache disorder and thus of benign etiology [23], is the most prevalent neurological symptom in the ED [15].

Conclusions regarding outcomes and impact of DAMA/PL on patient safety is limited because information about ED visits and admissions, in particular to other hospitals, or subsequent treatment in outpatient settings is lacking. Reasons for DAMA/PL were not recorded for every patient. There appear, however, to exist different, but interrelated, types of reasons for DAMA/PL. First, there are individual reasons such as personal or professional commitments perceived as conflicting with a hospital admission or preference of a different hospital. Such reasons, as well as expressions of anger and dissatisfaction, may mask a patient’s feelings of fear and helplessness related to hospital admission. The recognition of these factors and proactive communication guided by principles of shared decision-making between patients and physicians are among the strategies suggested to reduce or prevent premature and irregular patient discharges [8, 24]. Moreover, interventions such as increased rounding frequency of ED nurses and doctors [25, 26] or establishing patient advocates in the ED [27] improve patient satisfaction in the emergency room, which in turn has been shown to reduce rates of patients who leave without being seen or against medical advice [26].

Another patient-related factor impacting on the wish to leave may be related to the low rate of declined diagnostic procedures in DAMA/PL patients, particularly with regard to imaging as patients may not see any need for further work-up once a serious structural CNS pathology has been ruled out. While data regarding the reassuring effect of investigations are conflicting [28, 29], a positive influence of cranial imaging on patients’ levels of fear and anxiety has been noted [30].

Long waiting times represent a main reason to prematurely leave the ED [11] that certainly contains a patient-related aspect, but they also indicate structural insufficiencies in prehospital assessment in particular: in recent years, EDs have been dealing with increasing numbers of patients with neurological complaints [31], many of whom present with non-urgent complaints but nonetheless consider themselves in need of urgent medical evaluation [32]. Inappropriate utilization of ED resources promotes overcrowding and negatively impacts on both door-to-doctor times as well as ED length of stay. Consequently, a structured evaluation for treatment urgency for patients with neurological complaints is needed, all the more so because neurological symptoms are not adequately represented in current triage systems. Interdisciplinary EDs may benefit from a dedicated emergency neurology nurse overseeing patient management in co-operation with the neurologist on-call, and may, in particular, redirect patients with non-urgent complaints towards alternative care providers before they actually enter the ED. This may be particularly relevant for the conditions frequently encountered in DAMA/PL patients, i. e. headache and seizure in cases of previously known epilepsy, where the vast majority is of benign etiology [17], or no relevant diagnostic or therapeutic consequences ensue [33], respectively. Moreover, limited health literacy poses a relevant challenge for health practices and policies [34], maybe even more so in light of the complexities of many neurological disorders [35]. Among others, campaigns directed to patients and families to increase knowledge about alternative care providers from the ED may aid in improving demand management. It can be assumed that this also, through impacting on ED waiting times, will have an indirect influence on DAMA/PL rates. In sum, the investigation of DAMA/PL patients both in general as well as in particular patient populations, ideally informed by research into patient motivations for ED presentation, provides an opportunity to identify aspects requiring improvement on an institutional and a systemic level targeting demand management and patient direction and flow.

### Limitations

Our study has several limitations. To begin with, as a retrospective chart review, it critically rests on the completeness and accuracy of medical records. Particularly in PL patients, documentation is often incomplete. In DAMA patients, a more complete and detailed investigation into motivations for leaving would be informative. A further limitation concerns the conflation of DAMA and PL patients for parts of the analysis. DAMA patients left after an informed consent discussion but PL patients did not, so this may have an impact on the likelihood of readmission and risks associated with leaving the hospital. In addition, DAMA/PL patients left at different stages of the ED visit. Hence, it was not always clear whether a complete evaluation would have resulted in admission or discharge. We acknowledge this limitation by comparing DAMA/PL, i. e. all patients discharged irregularly, against all patients who were admitted or discharged. Moreover, discharge diagnoses will certainly be imprecise or tentative in those cases where patients left before ED work-up was complete. Finally, information regarding ED visits and admissions to other hospitals or treatments in outpatient settings and other outcome information is lacking. Accordingly, prospective investigations including follow-up assessment, e.g. via telephone interviews, would be desirable.

## Conclusions

We identified younger age and self-presenting mode of presentation as well as presentation with headache, seizures – in the majority of cases in a known history of epilepsy – or sensory deficits as being associated with irregular discharge of patients with neurological complaints from the ED. ED staff should be aware of these factors in order to apply adequate communicative and procedural strategies for the prevention of ED premature or against-medical-advice discharge. Long waiting times as the main reason for irregular leave from the ED point towards systemic insufficiencies in the pre-hospital assessment of patients with neurological complaints as well as a lack of alternative care providers.

## Data Availability

The datasets used and/or analysed during the current study are available from the corresponding author on reasonable request.

## Abbreviations

CT: Computed tomography
DAMA: Discharge against medical advice
ECG: Electrocardiogram
ED: Emergency department
ED: Emergency department
EEG: Electroencephalography
LP: Lumbar puncture
MRI: Magnetic resonance imaging
PL: Premature leave
